# High aortic pulse-wave velocity may be responsible for elevated red blood cell distribution width in overweight and obese people: a community-based, cross-sectional study

**DOI:** 10.5830/CVJA-2016-005

**Published:** 2016

**Authors:** Ibrahim Halil Altiparmak, Muslihittin Emre Erkus, Ozgur Gunebakmaz, Sezen Yusuf, Kaya Zekeriya, Recep Demirbag, Aydemir Kocarslan, Hatice Sezen, Ali Yildiz

**Affiliations:** Department of Cardiology, Faculty of Medicine, Harran University, Sanliurfa, Turkey; Department of Cardiology, Faculty of Medicine, Harran University, Sanliurfa, Turkey; Department of Cardiology, Faculty of Medicine, Harran University, Sanliurfa, Turkey; Department of Cardiology, Faculty of Medicine, Harran University, Sanliurfa, Turkey; Department of Cardiology, Faculty of Medicine, Harran University, Sanliurfa, Turkey; Department of Cardiology, Faculty of Medicine, Harran University, Sanliurfa, Turkey; Department of Cardiovascular Surgery, Faculty of Medicine, Harran University, Sanliurfa, Turkey; Department of Clinical Biochemistry, Faculty of Medicine, Harran University, Sanliurfa, Turkey; Department of Cardiology, Faculty of Medicine, Hacettepe University, Ankara, Turkey

**Keywords:** erythrocyte indices, vascular stiffness, overweight, obese

## Abstract

**Background:**

Obesity and overweight are risk factors for atherosclerosis. Red blood cell distribution width (RDW) is associated with subclinical cardiac diseases. The aim of this study was to investigate the association between RDW and aortic stiffness in overweight or obese subjects.

**Methods:**

A total of 101 overweight or obese subjects without overt cardiovascular disorders, and 48 healthy controls were enrolled. RDW, aortic pulse-wave velocity (PWV) and augmentation index 75 (Aix75) were evaluated. The case subjects were divided into two sub-groups according to PWV values; ≥ 10 m/s in group I, and < 10 m/s in group II. Bivariate correlation and multiple regression analyses (stepwise) were performed.

**Results:**

RDW and PWV were considerably increased in the case groups compared with the controls. RDW was significantly increased in group I compared with group II and the controls [median 12.0 m/s, interquartile range (IQR): 10.5–17.5; median 11.7 m/s, IQR: 10.2–14.2, and median 11.4 m/s, IQR: 9.6–15.5, p < 0.05, respectively]. Resting heart rate and age were higher in group I than group II (81 ± 11 vs 74 ± 12 beats/min and 41 ± 120 vs 36 ± 9 years, respectively, p < 0.05). Regression analyses revealed that while log-RDW, age and resting heart rate were independent predictors for aortic PWV, log-RDW was the most important predictor in the final model.

**Conclusions:**

RDW, resting heart rate and age independently predicted arterial stiffness, and RDW may be useful to provide an early recognition of subclinical atherosclerosis in overweight and obese individuals.

## Background

In addition to traditional risk factors, overweight and obesity are important risk factors for the development of atherosclerosis and cardiovascular events.^1^ Previous studies demonstrated that arterial stiffness (AS) was impaired in these populations.^2^ AS, which is a result of functional and structural disorders of the arterial wall, signifies end-organ damage and increased risk of cardiovascular events.^3^

Several indicators provide valuable information about AS. Among these, aortic pulse-wave velocity (PWV) and augmentation index occupy an important place. Many studies have demonstrated that aortic PWV is associated with subclinical coronary atherosclerosis, significant coronary artery disease, hypertension and kidney disease, and it has a predictive value for cardiovascular events.^3^-^6^

Red cell distribution width (RDW), part of a routine complete blood count, is a laboratory evaluation of the variability in the volume and size of circulating erythrocytes. It is usually used for a differential diagnosis of anaemia.^7^,^8^ Many recent studies have revealed relationships between high RDW levels and adverse cardiovascular conditions, such as heart failure,^9^ severity and complexity of coronary artery disease,^10^ coronary slow flow,^11^ isolated coronary artery ectasia,^8^ acute myocardial infarction,^12^ and lack of coronary collateral vessels in acute coronary syndromes.^7^ The exact mechanisms causing elevated RDW are uncertain in these clinical events. However, it has been asserted that inflammation and oxidative stress may be possible pathophysiological mechanisms underlying increased RDW levels in cardiovascular diseases.^11^

To our knowledge, the association of RDW with the markers of AS in overweight and obese individuals is unknown. The aim of the study was to determine whether there was any relationship between RDW and AS in this population.

## Methods

A total of 159 consecutive overweight or obese volunteers without overt cardiovascular disorders was enrolled as the case group and 48 healthy volunteers formed the control group in this community-based, cross sectional study (age between 18 and 75 years, mean 38 ± 11 and 37 ± 7 years, respectively). Fiftyeight of the case group who had systemic diseases, were on any medication, and consumed alcohol or smoked were excluded from the study.

**Fig. 1:**
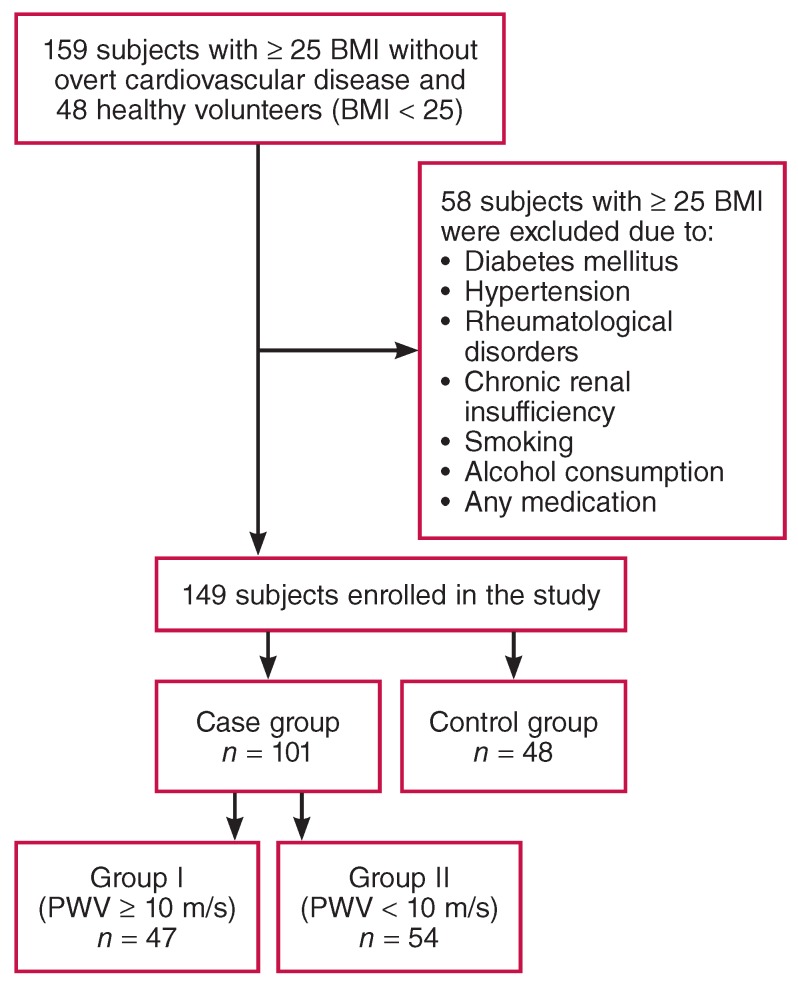
Diagram of the study design. BMI: body mass index; PWV: pulse-wave velocity.

The aortic PWV and aortic normalised augmentation index to 75 beats/min heart rate (Aix75) were measured in the remaining participants. The case subjects were divided into two groups based on aortic PWV values; those with aortic PWV ≥ 10 m/s were included in group I, and those with aortic PWV < 10 m/s were included in group II. The study design is shown in Fig. 1.

The study conformed to the recommendations of the Declaration of Helsinki on biomedical research involving human subjects. The study protocol was approved by the ethics committee and each participant provided written, informed consent.

## Measurement of RDW and AS

Samples of peripheral venous blood were drawn from the antecubital vein on admission after local antisepsis. Complete blood counts were measured using an autoanalyser (Sysmex K-1000, Block Scientific, USA) within five minutes of sampling. RDW (%) is one of the parameters automatically calculated during a full blood count. It is an index of the size distribution of red blood cells.

The following measurements were assessed on a TensioMed^TM^ arteriograph (TensioMed Ltd, Hungary): aortic PWV (m/s), aortic Aix75 (%), resting heart rate (beats/min), and systolic and diastolic blood pressures (mmHg). These parameters were measured according to the recommendations of the European Society of Hypertension for blood pressure and resting heart rate measurements.^13^

Subjects rested in the sitting position for at least five minutes and measurements were taken non-invasively using an appropriate cuff on the TensioMed^TM^ arteriograph. The choice of cuff size (small, medium and large) was automatically determined by the arteriography according to the arm circumference, and the jugulum symphysis size reflects the interval from the aortic root to the bifurcation.

Arteriography perceives brachial artery pulsations based on the oscillometric principle, and assesses parameters of AS by analysing pulse-wave forms. Aortic PWV is determined by calculating the travelling time of the pulse wave between two reference points.^14^ As there is a linear relationship between heart rate and Aix, the Aix is standardised to a heart rate of 75 beats/ min (Aix75). Aix (%) represents the ratio of reflected wave to primary wave, which moves from heart to tissues. It is inversely associated with arterial or aortic compliance.^15^

All parameters were measured by the investigators in the morning between 8:00 and 10:00, after 12 hours of fasting, and the average of at least three measurements was used.

## Statistical analysis

All analyses were done using SPSS version 20.0 (IBM Corporation, USA). The parameters were expressed as numbers (percentage) for categorical data, mean ± SD for parametric data, and median with interquartile range (IQR) for non-parametric data. We used the one-sample Kolmogorow–Smirnow test to assess normality of the data. The Student’s t-test (for parametric data) and the Mann– Whitney U-test (for non-parametric data) were used to compare variables between the two groups. Also, ANOVA and the Kruskal– Wallis tests were used for comparisons between the three groups (for parametric and non-parametric variables, respectively). To test gender differences between the groups, the chi-squared test or Fisher’s exact test, where appropriate, were used.

Fisher’s exact test, where appropriate, were used.Statistical significance between the variables was set at p < 0.05. We used bivariate correlation analysis to determine the correlation between statistically significant variables. Pearson’s correlation was used for data with normal distributions, and Spearman’s correlation was used for data with a skewed distribution.

After definition of the factors that were associated with aortic PWV in bivariate analysis, independent predictors for estimation of aortic PWV were determined using multiple linear regression analyses with stepwise exclusion of these factors, using a criterion of p < 0.05 for retention of factors in the model. When evaluating RDW with skewed distribution in these analyses (correlation and regression), the variable was log-transformed (ln) and this logarithmic value was entered into the correlation and regression models.

## Results

**Fig. 2:**
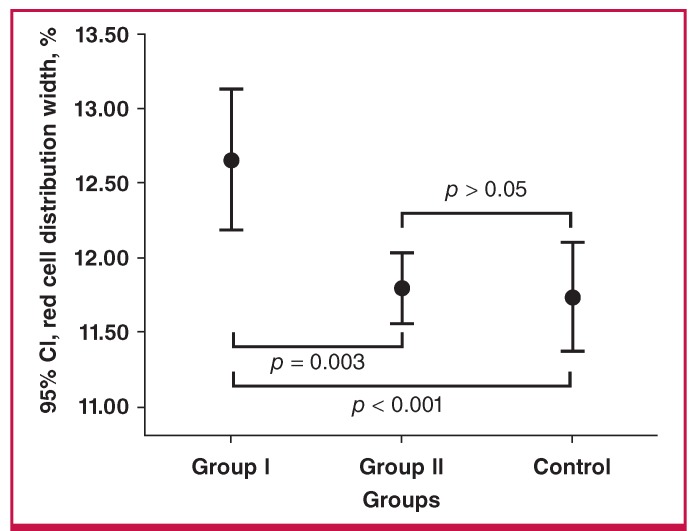
The error bar graph showing RDW difference between the groups. RDW: red cell distribution width.

The clinical and laboratory parameters of the two groups are presented in Table 1. Aortic PWV, RDW, body mass index (BMI) and waist–hip ratio were considerably higher in the case groups than the controls (p < 0.05) (Table 1). In subgroup analyses, RDW was significantly increased in group I compared with group II and the controls (p = 0.003 and p < 0.001, respectively), although there was no statistically significant change between group II and the controls (p > 0.05) (Table 2, Fig. 2).

**Table 1:** Comparison of case group with control group

*Parameters*	*Case group (n = 101)*	*Control group (n = 48)*	*p-value*
Male, n (%)	72 (71)	32 (67)	0.566
Age, years	38 ± 11	37 ± 7	0.564
Body mass index, kg/m^2^	28.8 ± 3.3	22.4 ± 1.6	< 0.001
Waist–hip ratio	0.91 ± 0.10	0.82 ± 0.07	< 0.001
Systolic blood pressure, mmHg	121 ± 9	120 ± 8	0.655
Diastolic blood pressure, mmHg	78 ± 5	77 ± 6	0.110
Resting heart rate, beats/min	77 ± 11	76 ± 12	0.719
Aortic pulse wave velocity, m/s	9.9 ± 2.0	9.1 ± 2.3	0.027
Aortic augmentation index 75, %	19 ± 11	17 ± 12	0.275
Fasting glucose, mg/dl	90 ± 5	88 ± 6	0.056
(mmol/l)	(5 ± 0.28)	(4.88 ± 0.33)	
Urea, mg/dl	28 ± 5	26 ± 5	0.058
Creatinine, mg/dl	0.80 ± 0.08	0.78 ± 0.08	0.175
(mmol/l)	(70.72 ± 7.07)	(68.95 ± 7.07)	
Alanine aminotransferase, U/l	26 ± 5	24 ± 4	0.059
Triglycerides, mg/dl	150 ± 19	149 ± 21	0.919
(mmol/l)	(1.70 ± 0.21)	(1.68 ± 0.24)	
Total cholesterol, median (IQR), mg/dl	177 (128–244)	176 (116–227)	0.201*
(mmol/l)	[4.58 (3.32–6.32)]	[4.56 (3.0–5.88)]	
LDL cholesterol, mg/dl	109 ± 19	102 ± 17	0.154
(mmol/l)	(2.82 ± 0.49)	(2.64 ± 0.44)	
HDL cholesterol, median (IQR), mg/dl	42 (33–52)	42 (38–53)	0.184*
(mmol/l)	[1.09 (0.85–1.35)]	[1.09 (0.98–1.37)]	
RDW, median (IQR), %	11.9 (10.2–17.5)	11.4 (9.6–15.5)	0.005*
White blood cell, 10^3^/μl	8.1 ± 1.6	7.6 ± 1.6	0.086
Haematocrit, %	44 ± 5	43 ± 5	0.219

IQR: interquartile range; HDL: high-density lipoprotein; LDL: low-density lipoprotein; RDW: red cell distribution width.

*p-value of Mann–Whitney U-test.

**Table 2:** Comparison of variables between the three groups

*Parameters*	*Group I (n = 47)*	*Group II (n = 54)*	*Control (n = 48)*
Males, n (%)	34 (72)	34 (63)	32 (67)
Age, years	41 ± 12^a^	36 ± 9	37 ± 7
Body mass index, kg/m^2^	29.1 ± 3.4^b^	28.3 ± 2.9^c^	22.4 ± 1.6
Waist–hip ratio	0.94 ± 0.10^b^	0.90 ± 0.09^c^	0.82 ± 0.07
Systolic blood pressure, mmHg	122 ± 8	120 ± 10	120 ± 8
Diastolic blood pressure, mmHg	80 ± 5	78 ± 6	77 ± 6
Resting heart rate, beats/min	81 ± 11	74 ± 12^a^	76 ± 12
Aortic pulse wave velocity, m/s	11.6 ± 1.4^a,b^	8.4 ± 0.9	9.0 ± 2.3
Aortic augmentation index 75, %	23 ± 13	17 ± 9	17 ± 12
Fasting glucose, mg/dl	90 ± 5	89 ± 6	88 ± 6
(mmol/l)	(5 ± 0.28)	(4.94 ± 0.33)	(4.88 ± 0.33)
Urea, mg/dl	28 ± 5	28 ± 4	26 ± 5
Creatinine, mg/dl	0.81 ± 0.06	0.80 ± 0.09	0.78 ± 0.08
(mmol/l)	(71.60 ± 5.30)	(70.72 ± 7.96)	(68.95 ±7.07)
Alanine aminotransferase, U/l	25 ± 5	27 ± 4	24 ± 4
Triglycerides, mg/dl	150 ± 19	148 ± 17	149 ± 21
(mmol/l)	(1.70 ± 0.21)	(1.67 ± 0.19)	(1.68 ± 0.24)
Total cholesterol, median (IQR), mg/dl	177 (128–244)	178 (136–223)	176 (116–227)
(mmol/l)	[4.58 (3.32–6.32)]	[4.61 (3.52–5.78)]	[4.56 (3.0–5.88)]
LDL cholesterol, mg/dl	106 ± 18	108 ± 18	102 ± 17
(mmol/l)	(2.75 ± 0.47)	(2.80 ± 0.47)	(2.64 ± 0.44)
HDL cholesterol, median (IQR), mg/dl	41 (34–52)	42 (33–48)	42 (38–53)
(mmol/l)	[1.06 (0.88–1.35)]	[1.09 (0.85–1.24)]	[1.09 (0.98–1.37)]
RDW, median (IQR), %	12.0 (10.5–17.5)^a,b^	11.7 (10.2–14.2)	11.4 (9.6–15.5)
White blood cell, 10^3^/μl	8.3 ± 1.7	7.9 ± 1.6	7.6 ± 1.6
Haematocrit, %	44 ± 7	45 ± 4	43 ± 5

Group I: pulse-wave velocity ≥ 10 m/s; group II: pulse-wave velocity < 10 m/s; IQR: interquartile range; HDL: high-density lipoprotein; LDL: low-density lipoprotein; RDW: red cell distribution width.

^a^p < 0.05 between group I and II; ^b^p < 0.05 between group I and control; ^c^p < 0.05 between group II and control.

Aortic PWV was significantly higher in group I than in group II and the controls (p < 0.001 for both); however, it was similar in group II and the controls (p > 0.05). Although age was similar in group II and the controls (p > 0.05), it was considerably higher in group I than in group II (p = 0.046). While BMI and waist–hip ratio were significantly higher in groups I and II than in the controls, as expected (p < 0.001), there was no statistically significant difference between groups I and II (p > 0.05) (Table 2).

**Fig. 3:**
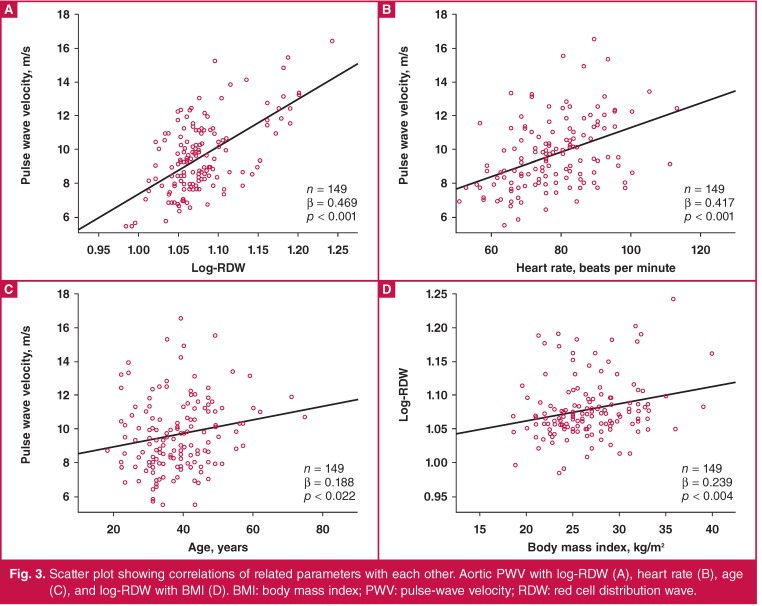
Scatter plot showing correlations of related parameters with each other. Aortic PWV with log-RDW (A), heart rate (B), age (C), and log-RDW with BMI (D). BMI: body mass index; PWV: pulse-wave velocity; RDW: red cell distribution wave.

Bivariate correlation analyses showed positive correlations of log-RDW, resting heart rate, age and BMI with aortic PWV (p < 0.001, p < 0.001, p = 0.022, and p = 0.007, respectively) (Table 3, Fig. 3A-C). Also, there was a positive correlation between log-RDW and BMI (Fig. 3D). However, multiple regression analyses (stepwise) using variables with significant correlation revealed that log-RDW, resting heart rate and age independently predicted aortic PWV. Of these, log-RDW was a stronger predictor than age and resting heart rate in the final model (Table 3).

**Table 3:** Bivariate correlations and regression analyses (stepwise) of aortic PWV with related parameters

			*Stepwise regression analyses*					
	*Bivariate correlation*		*Model I (R^2^=0.304)*		*Model II (R^2^=0.456)*		*Model III (R^2^=0.492)*	
*Parameters*	R	p	β	p	β	p	β	p
Aortic PWV								
Log-RDW	0.469	< 0.001	0.551	< 0.001	0.506	< 0.001	0.457	< 0.001
Resting heart rate	0.417	< 0.001			0.393	< 0.001	0.421	< 0.001
Age	0.188	0.022					0.196	0.003
BMI	0.223	0.007						
WHR	0.128	0.174						

For regression analyses of aortic PWV; predictors in model I: log-RDW; predictors in model II: log-RDW and resting heart rate; predictors in model III: log- RDW, resting heart rate and age.

Aix75: augmentation index 75; BMI: body mass index; PWV: pulse-wave velocity; RDW: red cell distribution width; WHR: waist–hip ratio.

## Discussion

The main findings of this community-based, prospective study were: (1) the subjects with high aortic PWV had advanced age, increased RDW and increased resting heart rate; (2) these parameters were positively correlated with aortic PWV; and (3) log-RDW, resting heart rate and age were independent predictors for increased aortic stiffness (aortic PWV ≥ 10 m/s), and in the final model the strongest predictor among them was log-RDW.

We know that increased body fat mass can lead to atherosclerotic vascular diseases.^2^ If there is no evidence of cardiovascular disease or disorders such as hypertension, hyperlipidaemia and diabetes in overweight or obese individuals, they are not treated with medication, but lifestyle changes are suggested.^16^ Despite this, cardiovascular events appear more frequently in the obese population than in individuals with a lower BMI.^1^

In this study, we speculated that RDW may be used to determine persons at high risk for atherosclerosis among obese and overweight individuals without overt cardiovascular disorders. Thus individuals who are prone to atherosclerosis could more easily be identified using RDW and more closely followed up.

RDW, which is usually overlooked and almost only used in the differential diagnosis of anaemia in daily practice, is typically raised in some cardiac and non-cardiac conditions, such as coronary artery disease, peripheral artery disease, acute coronary syndromes, heart failure, malnutrition, neoplastic metastases to bone marrow and inflammatory bowel disease.^7^,^17^-^19^

RDW generally reflects ineffective red cell production or their increased rate of destruction. However, it has been demonstrated that oxidative stress and chronic inflammation also cause elevated RDW. By destruction of the erythrocytes, haemolytic conditions lead to elevation of RDW, and impaired erythropoiesis, haemoglobinopathies, folic acid or cyanocobalamin deficiency, and iron deficiency also cause raised RDW.^19^,^20^

On the other hand, inflammatory cytokines have an effect on iron metabolism and the production or activity of erythropoietin.

They also impair the erythrocyte membrane and maturation of red blood cells. As a result, they lead to the release of juvenile erythrocytes from bone marrow into the circulation.^21^ Additionally oxidative stress can affect the production of red blood cells. These cells have tremendous antioxidant capacity and are a primary oxidative sink, nevertheless, they are susceptible to oxidative damage.

The oxidative stress associated with many clinical conditions brings about raised RDW.^8^ Activation of neurohumoral mediators (such as angiotensin II and the sympathetic system hormones) as well as inflammation and oxidative stress stimulate abnormal erythropoiesis. Therefore the survival of red blood cells is diminished on account of ineffective erythropoiesis.^7^,^21^ All of these conditions can be observed in atherosclerotic cardiovascular disease, leading to elevation of RDW.

In the present study, we aimed to investigate whether RDW had an association with AS, which is an indicator of subclinical atherosclerosis. We hoped to recognise atherosclerosis at an early stage using RDW level, which is a routine parameter of a full blood analysis in most patients. Because RDW could be affected by many clinical conditions, we included healthy volunteers with a high BMI in the study. Those with low haematocrit levels or who had undergone blood transfusions in the previous six month were excluded. We did not investigate whether there were iron, folic acid or cyanocobalamine deficiencies in the subjects, since the mean haematocrit level of the study population was normal.

The results of this study support our hypothesis, and indicate the strong positive correlation between log-RDW and aortic PWV. Also, age, resting heart rate and BMI were positively correlated with aortic PWV. However, multiple regression analysis suggested that log-RDW, resting heart rate and age were independent predictors for aortic PWV.

It is known that elasticity of the arteries is impaired with advanced age.^22^,^23^ Similarly, it has been reported that increased resting heart rate is associated with aortic stiffness and atherosclerosis.^24^,^25^ Our study supports these results. However, an interesting result of our study was that RDW was the most important independent predictor for aortic PWV in the stepwise regression analyses.

In clinical practice, we believe that RDW and resting heart rate, especially with advanced age, can indicate AS or subclinical atherosclerosis in obese and overweight individuals. Therefore, these patients may be identified at an early stage of atherosclerosis and medically treated earlier and more aggressively, before the development of any symptoms of atherosclerotic disease.

Aortic PWV, which is the travelling time of the pulse wave from one point to another, indicates arterial stiffness, and high values reflect atherosclerotic cardiovascular disease with or without symptoms.^4^,^6^,^26^ Atherosclerotic disease may be determined by various symptoms or acute events; however, subclinical atherosclerosis is not be easily recognised. Therefore, some determinants of AS, such as aortic PWV and Aix75, have been used to detect subclinical atherosclerosis.

Aortic PWV is the gold standard to evaluate AS and cardiovascular risk, as has been shown in many studies.^13^,^16^,^25^,^26^ A number of recently published studies have demonstrated elevated aortic PWV in several cardiovascular disorders. Kullo et al. showed that aortic PWV was related to subclinical coronary atherosclerosis, detected by the presence of coronary artery calcium using computed tomography.^4^ Another study by Catalano et al. pointed out that augmentation index in patients with peripheral artery disease, which is another parameter of AS, was higher than in the normal population.^27^ Our study showed no changes in aortic Aix75 between the case subjects and controls, unlike aortic PWV.

Obesity is an important risk factor for various health problems, including atherosclerosis. It has recently been demonstrated that people using high-fat dairy products had a higher aortic PWV and carotid media thickness compared with those using low-fat dairy products.^28^ Vayá et al. and Fujita et al. reported an elevated RDW in obese subjects, and our results are compatible with these.^29^,^30^ However, previous studies asserted that different causes might be responsible for this elevation. Among them, inflammation and hyposideraemia were blamed as responsible mechanisms.^29^,^30^ There is no consensus on the pathogenesis of the rise in RDW in obese and overweight populations.

We also demonstrated a raised RDW and PWV in obese and overweight persons compared with the healthy controls. However, when the case group was divided into two subgroups according to aortic PWV, the RDW of the overweight and obese subjects with low aortic PWV was similar to that of the controls. Conversely, those with high aortic PWV had a considerably elevated RDW compared with the controls and the other group (obese and overweight with low PWV).

Also, although there was a linear correlation between BMI and RDW as well as aortic PWV, stepwise regression analyses showed that log-RDW was an independent predictor in the final models for the estimation of aortic PWV. These results indicated that impaired aortic elastic properties with high aortic PWV may contribute to increased RDW in overweight and obese patients, apart from the previously stated reasons.

There are some limitations of our study. The main limitation is the relatively small sample size. Another is that iron, folic acid and cyanocobalamin levels were not determined in the participants. However, volunteers with a haematocrit lower than 35% were not included in the study. Lastly, we did not evaluate adipocytokines, such as leptin, adiponectin, resistin, chemerin and visfatin, knowing their relationship with atherosclerosis and angiogenesis.

## Conclusion

This study indicated that raised aortic stiffness may be responsible for elevated RDW values in obese and overweight persons. Therefore, RDW may help to provide an early recognition of atherosclerosis using a simple test instead of more sophisticated devices, particularly if evaluated together with resting heart rate in the older population.
